# The opportunities and challenges posed by the new generation of deep learning-based protein structure predictors

**DOI:** 10.1016/j.sbi.2023.102543

**Published:** 2023-02-18

**Authors:** Mihaly Varadi, Nicola Bordin, Christine Orengo, Sameer Velankar

**Affiliations:** 1 Protein Data Bank in Europe, European Molecular Biology Laboratory, European Bioinformatics Institute (EMBL-EBI), Welcome Genome Campus, Hinxton, Cambridge, CB10 1SD, UK; 2 Institute of Structural and Molecular Biology, University College, London, London, WC1E 6BT, UK

**Keywords:** Protein Structure Predictions, Deep learning, Structural biology, Structural Bioinformatics.

## Abstract

The function of proteins can often be inferred from their three-dimensional structures. Experimental structural biologists spent decades studying these structures, but the accelerated pace of protein sequencing continuously increases the gaps between sequences and structures. The early 2020s saw the advent of a new generation of deep learning-based protein structure prediction tools that offer the potential to predict structures based on any number of protein sequences. In this review, we give an overview of the impact of this new generation of structure prediction tools, with examples of the impacted field in the life sciences. We discuss the novel opportunities and new scientific and technical challenges these tools present to the broader scientific community. Finally, we highlight some potential directions for the future of computational protein structure prediction.

## Introduction

One of the fundamental approaches to understanding the function of proteins is to investigate their three-dimensional structures [1]. The most reliable approach to determining the structure of proteins involves time-consuming and expensive experimental techniques. Based on structures archived in the Protein Data Bank (PDB), the most prevalent experimental method is X-ray crystallography, followed by electron microscopy (EM) [2]. While these methods can yield high resolution and high-quality protein structures, the associated effort and costs make it impossible for structural biologists to keep up with the ever-increasing number of known protein sequences. Indeed, the gap between protein sequences and their structures has grown by orders of magnitude over the past decade [3].

Protein structures are determined solely by their amino acid sequences, challenging researchers and scientific software developers to design algorithms to accurately predict protein structures from sequence data [4]. Computational protein structure prediction tools have been around for decades, but the early 2020s saw a huge step forward in terms of the accuracy of models [5]. The unprecedented quality of models predicted by Alpha-Fold 2.0 during the 14th Critical Assessment of Structure Prediction competition, followed by the release of an improved version of RoseTTAFold, made accurate protein structure prediction tools available to the broad scientific community [6–8]. These models can now match the accuracy of experimentally determined structures and sometimes even surpass them, as observed in an extensive comparison of NMR-based protein structures and predicted models [9,10]. It is important to note, that while these tools do not require template structures, they do rely on sufficiently deep multiple sequence alignments (MSA). Shallow MSAs lead to poor model quality, reflected in low local confidence scores. These algorithms provide confidence metrics such as the pLDDTscore, which corresponds to the model’s prediction of its score on the local Distance Difference Test, and the predicted aligned error (PAE), which gives information on the confidence of the relative position of residue pairs in the model. Since the release of these algorithms, research groups have performed rigorous validation and assessment of predicted coordinates and pLDDT confidence scores against various classes of proteins, such as transmembrane proteins, centrosomal and centriolar proteins, and whole proteomes, with only the validation of PAE data yet to be comprehensive [11–13].

In 2022, over 214 million predicted protein structures became available in the AlphaFold Protein Structure Database (AlphaFold DB), covering most of the sequences in the UniProt database [14]. Access to predicted protein structures on this scale made structural data available to a broader audience than ever before. Researchers with no prior experience in protein modelling can now use these models to tackle challenging biological problems, noting that familiarity with model confidence metrics is still essential to making robust interpretations.

In this review, we give an overview of how the massive amount of predicted protein structures and the underlying open-source algorithms impact the life sciences. We discuss new opportunities and new challenges posed by these significant developments. Finally, we speculate about the directions protein structure prediction might move towards next.

### The impact of high-accuracy protein structure models

**Figure 1:**
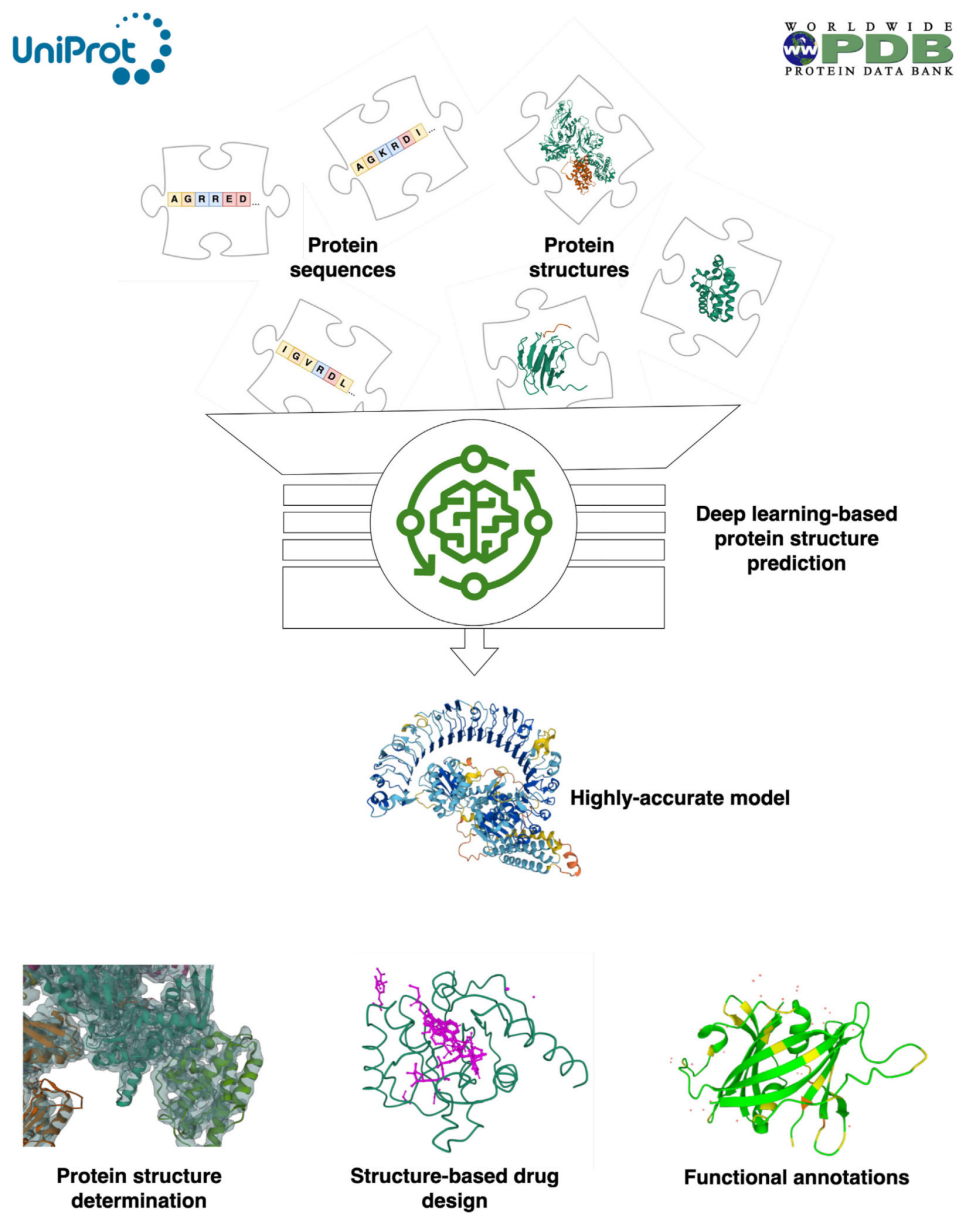
AnAn overview of deep learning-based protein structure prediction workflows The new generation of protein prediction tools used protein sequence data from the UniProt database and protein structures from the PDB to train their models. These tools can provide predicted structures for virtually any protein sequence. This benefits protein structure determination efforts by fitting against experimental data and provides input to structure-based drug design pipelines and structure-based functional annotation software.

The new generation of protein prediction tools required data from the public protein sequence and protein structure resources to train their algorithms. Predicted models now benefit structure determination efforts, structure-based drug design and structural bioinformatics analysis on a scale that was impossible before (Figure 1) [14].

Over the past decades, structural biologists solved over 190,000 macromolecular structures and made them publicly available through the PDB archive [2]. Now, tools such as AlphaFold help scientists predict protein structures that proved too elusive in the past. Predicted protein structures are now routinely used to assist in crystallographic phasing by molecular replacement [15,16] and to fit predictions against electron-microscopy maps [17]. Similar synergistic approaches that combine experimental data and predictions have helped determine the structure of challenging molecular machines, such as the nuclear pore complex [18,19].

Predicted models are not replacing experimentally determined protein structures, especially structures of large macromolecular assemblies, but they have affected specific software and data processing pipelines. For example, the Diamond Light Source, the UK’s national synchrotron, has AlphaFold configured on-site to help researchers combine predicted models with the X-ray diffraction data they obtain as part of the downstream data processing pipeline. Indeed, synchrotrons, EM facilities and bioinformatics facilities now frequently host an instance of AlphaFold on-site. Another emerging practice is using predictions to design crystallisation constructs by identifying and excluding longer flexible segments, improving the chance of successful crystallisation [20]. In another application, predicted models help identify potentially interesting post-translational modification (PTM) sites. PTM sites are generally found on accessible, flexible regions of proteins, and AlphaFold models can help locate such regions. This approach effectively filters the number of potential PTM sites and helps researchers focus on the experimental evaluation of more likely candidate sites [21].

The large-scale application of deep learning-based protein structure modelling highlighted the prevalence of intrinsically disordered regions in every modelled proteome [22]. While initial reports suggested that the confidence measure of AlphaFold, the pLDDT score [12], strongly correlates with intrinsic disorder propensity, recent studies show a more complex relationship [23]. Based on large-scale analyses, pLDDT scores lower than 50 are caused either by genuinely poor predictions due to shallow multiple sequence alignments or negatively correlated with intrinsic disorder propensities. However, this correlation seems to hold only for so-called entropic chains and flexible linkers [24]. In the case of disordered regions that adopt stable tertiary structures through binding-induced folding, AlphaFold generally predicts the bound forms with high pLDDT scores [25].

The availability of experimental structures in the PDB led to the birth and steady growth of protein domain classification efforts such as CATH, SCOP, ECOD, SCOPe and SCOP2 [26–29], where protein domains are identified and classified according to their evolutionary history. The growth of these resources always depended on the growth of the PDB, and initiatives such as Gene3D and Pfam [30,31] aimed to increase the structural coverage of the sequence space by obtaining domain assignments using Hidden Markov Models (HMM) matches created from protein domains. AlphaFold dramatically changed the protein domain landscape, as millions of domain sequences became potentially well-modelled domain structures. While a boon for many scientists studying proteins without available structures in the PDB, the sheer size of the data and the potential consequences of basing further research on less-than-optimal models require careful vetting. For instance, 700,000 putative CATH domains were identified in the initial AlphaFold DB release of 21 model organisms, but filtering based on model quality and disordered regions reduced this number by 49% [32].

In addition to model quality considerations, predicted structures generally lack contextual molecules, which may cause inaccurate interpretations. For example, tools such as P2Rank rely on physicochemical attributes to identify potential binding sites. The absence of co-factors, ions and other small molecules in AlphaFold models can influence its behaviour [33]. Data resources such as AlphaFill help address this limitation by expanding AlphaFold models with cofactors, ions and ligands [34].

### New challenges posed by the scale of the available predicted structures

The dataset of 214 million predictions in the AlphaFold DB [14] immensely increased the coverage of the protein sequence space with protein structures and posed new challenges and opportunities in the fields of structural biology and structural bioinformatics. Analytical software optimised to process hundreds of thousands of protein structures may struggle to run efficiently on a much larger dataset. Even data retrieval of a custom data set to start larger scale analysis is not trivial.

While AlphaFold DB provides archive files (TAR files) for 48 proteomes and the Swiss-Prot data set, these are subsets of the data and have their own limitations. Specifically, the archive files only contain the atomic coordinates in compressed PDB and mmCIF files, but the equally important predicted aligned error (PAE) data is missing. The PAE data contains information about the confidence in the relative position of residue pairs. Without this information, it is impossible to determine if the position of two seemingly adjacent regions or domains in a predicted AlphaFold structure can be considered reliable.

While the PDB, mmCIF and PAE data can all be downloaded from the AlphaFold DB prediction pages, it is not a very efficient approach when collating a custom, large data set. To help address this, the complete dataset is made available on the Google Cloud Public Datasets platform. This allows users to retrieve the complete dataset (~23 terabytes, ~1 million TAR files) and to query the database for assembling and downloading large sets of predictions.

The availability of millions of predicted structures raises challenges ranging from data storage to identifying remote homologs and ways to traverse these new large swaths of structure space quickly. State-of-the-art methods for homology annotation of uncharacterized domains before the early 2020s relied on local alignment tools such as BLAST [35], searches against HMM libraries such as HMMER3 [36] and HHsuite [37] if only the sequences were available, or using accurate but very slow structural aligners like DALI, SSAP, TMalign, CE [38–41]. While still valuable, HMMs struggle to detect remote homologs, and using structure alignments is unfeasible with the sheer amount of structures available. Fortunately, the release of AlphaFold DB coincided with new language models that were successfully trained on proteins, and multiple new predictors based on embeddings from these protein language models were created, tested and validated in various scenarios and were found to outperform established tools for homology detection (including HMMs) [42,43], disorder prediction [44] and ligand-binding prediction [33,45,46]. In the case of AlphaFold-derived protein domain models, they identified in the first release of AlphaFold a correct CATH homologous superfamily for 8% of domains that were elusive to Hidden Markov Models.

Predicted domain assignments to homologous super-families require validation by structural comparisons against known homology domains. Using current structural aligners based on double dynamic programming such as SSAP or DALI isn’t a feasible solution due to the amount of AlphaFold-derived models. Almost concurrently with the first release of AlphaFold Database, Foldseek - a new, ultra-fast structural aligner by van Kempen and colleagues, was released with comparable accuracy to TMalign while being over 20,000x faster [47].

Combining embeddings-based predictions for homologous superfamily assignments and their validation using Foldseek opened the gates to large-scale annotations of protein domains across structure space. In addition to being applicable without requiring MSAs, embedding-based approaches have the added benefit of working on unlabelled data which could ease its application for tasks such as ligand binding prediction.

## Conclusion and future perspectives

Having unrestricted access to millions of predicted protein structures enables new and innovative research. While posing new challenges to existing scientific software, the amount of new structural data opens up many opportunities in several fields of the life sciences.

Predicting whole assemblies is perhaps the new frontier since these are the functional units in many biological processes. Indeed, accurate models for the whole human interactome may soon be within reach [48,49]. Shortly after the release of AlphaFold and RoseTTAFold, researchers experimented with adopting these algorithms to predict assemblies with some success. Concurrently, a team at DeepMind created a specialised version of AlphaFold, AlphaFold-Multimer, which achieved relatively good accuracy [50]. One could expect that the emphasis will shift to modelling assemblies, and the state-of-the-art algorithms will compete in the Critical Assessment of Prediction of Interactions (CAPRI) and CASP [51].

It would be similarly impactful to develop more accurate tools for modelling interactions between proteins and small molecules [52]. The availability of a reliable molecular docking algorithm based on advanced AI technologies could revolutionise the field of structure-based drug discovery and accelerate medical research [53].

Creating AI tools that can provide a window into the dynamic nature of proteins is another potential direction [54]. While AlphaFold already demonstrated the prevalence of structurally flexibly regions in many proteomes, these models only provide single snapshots from all the possible conformations [55]. Modelling biologically relevant conformational ensembles would open up new opportunities in understanding the biological function of many proteins and could allow drug discovery projects to target intrinsically disordered regions, which is notoriously challenging [56].

Other applications of AI-based structure prediction algorithms could include modelling the structural effects of post-translational modifications, the conformational consequences of mutations and variants, and applications in the field of protein design, but it is important to note that the current versions of popular tools like AlphaFold cannot predict the structural consequences of mutations [57–60].

The arrival of the new generation of accurate protein structure prediction tools is a transformative time for structural biology, structural bioinformatics, drug discovery and many other fields of the life sciences. While these tools apparently excel at their tasks, within certain limitations, the Research and innovation have been accelerated, and a new era of discovery through the application of advanced AI technologies has started.

## Data Availability

No data was used for the research described in the article.
